# Asystole during Suspension Laryngoscopy: Case Report, Literature Review, and Prophylactic Strategies

**DOI:** 10.1155/2020/9260564

**Published:** 2020-01-22

**Authors:** Arthur Justi Cassettari, Érica Cristina Campos e Santos, Graziela Oliveira Semenzati, Agrício Nubiato Crespo

**Affiliations:** ^1^Otorhinolaryngology Resident at the Department of Otorhinolaryngology, Faculty of Medical Sciences, State University of Campinas (UNICAMP), Campinas, São Paulo, Brazil; ^2^Laryngology Fellowship at the Department of Otorhinolaryngology, Faculty of Medical Sciences, UNICAMP, Campinas, Brazil; ^3^Otolaryngologist at the Department of Otorhinolaryngology, Faculty of Medical Sciences, UNICAMP, Campinas, Brazil; ^4^Head of Laryngology Service and Head of Department of Otorhinolaryngology, Faculty of Medical Sciences, UNICAMP, Campinas, Brazil

## Abstract

Transoral laryngeal procedures are considered minimally invasive but may trigger important complications such as severe bradycardia and even asystole mediated by vagal reflex. The literature on this subject is rare. This article aims to review the literature, explain associated mechanisms, establish prophylactic strategies, and highlight the importance of intraoperative safety protocols.

## 1. Introduction

Direct suspension laryngoscopy was first described by Killian in 1911. The technique is based on the use of specific laryngoscopes, suspended by a pectoral support, which allows for bimanual handling of the larynx during the procedure. With the advent of ancillary equipment such as the surgical microscope, carbon dioxide (CO_2_) laser, and endoscopes, the procedure became predominant for phonosurgeries and tumor resections, replacing open and more invasive techniques.

Although the technique is minimally invasive, potential complications, inherent to the method, are described. Most are of lower severity, such as dental, esophageal, tracheal, or lingual paraesthesia due to compression. The usual cardiac reactions, secondary to airway manipulation during direct laryngoscopy, are increased heart rate and blood pressure, which occur due to sympathetic reflex and release of catecholamines [1].

Bradycardia or, in extreme cases, asystole caused by suspension laryngoscopy is well described in children [2] but is uncommon in healthy adult patients.

This complication is documented in patients with severe cardiac disease or history of previous trauma with intracranial hypertension [3, 4]. This review illustrates the case of a patient without such comorbidities who underwent suspension laryngoscopy and evolved with asystole.

## 2. Case Report

**Figure 1:**
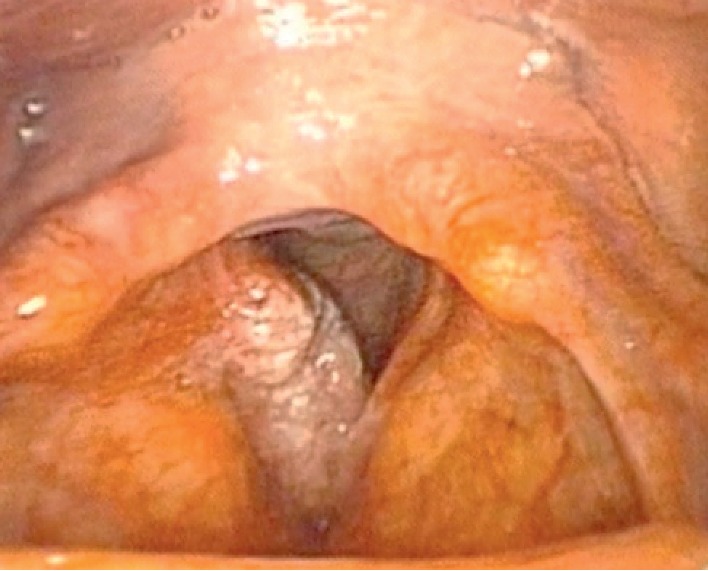
Vegetative lesion on the right vocal fold.

A 55-year-old male of 79 kg, with a history of controlled hypertension and smoking for 17 pack-years that was stopped five years ago, presented with progressive dysphonia for 18 months, characterized by moderate roughness and mild breathiness. GRBASI Scale graded him G2-R2-B1-A0-S0-I0. In videolaryngoscopy, there was a vegetative lesion on the right vocal fold suggestive of carcinoma, of stage T2N0Mx (Figure 1). It was proposed to perform a cordectomy using a CO_2_ laser by direct laryngoscopy, guided by frozen section analysis.

Cardiopulmonary physical examination and preoperative laboratory tests revealed no abnormalities. The electrocardiogram showed sinus cardiac rhythm, with a heart rate of 75 bpm, and the preoperative cardiological evaluation based on Lee's Revised Cardiac Risk Index was Class II. Spirometry was within the parameters of normality. The patient was classified by the pneumology team to have medium surgical risk and by anesthesiology as ASA Class II and Mallampati Class II.

During the anesthetic induction, the patient had a regular heart rate of 90 bpm and blood pressure of 130 × 80 mmHg. Continuous inhalation of sevoflurane and intravenous (IV) bolus injection of fentanyl (5 mcg/kg), propofol (2 mg/kg), and rocuronium (0.75 mg/kg) were administered. The patient was intubated with an endotracheal tube suitable for the use of CO_2_ laser, no. 4.5.

A biopsy was performed for frozen section analysis using direct laryngoscopy, uneventfully. During the 40 minutes of waiting for the anatomopathological result, the patient remained without laryngeal stimuli and received no other anesthetic drugs. Then, the procedure was restarted using the Bouchayer laryngoscope, with great difficulty for adequate exposure of the anterior commissure. At this time, a new bolus injection of rocuronium (0.25 mg/kg) was administered.

During the attempt for better laryngeal exposure, the anesthetic team reported bradycardia (30 bpm). The laryngoscope was immediately removed, and 1 mg of IV atropine was infused. Despite the measures, there was progression to asystole, and cardiopulmonary resuscitation (CPR) was promptly initiated. The patient's pulse returned after 2 cycles of CPR and 1 mg of IV adrenaline. After stabilization, the procedure was suspended and the patient was referred to the intensive care unit, where neurological and cardiopulmonary causes for the event were excluded.

During the hospitalization period, the patient evolved satisfactorily and did not present any neurological sequelae.

## 3. Discussion

In 1986, Wenig et al. [5] evaluated the cardiac complications of suspension laryngoscopy in 100 adult patients, 48 of whom had high previous cardiac risk. The main cardiac complications presented were increased blood pressure and tachycardia. None of the patients had severe bradycardia or asystole.

It is known that the main mechanism for the induction of severe bradycardia or asystole during suspension laryngoscopy is the exacerbated vagal response via parasympathetic fibers from the vagus nerve to the sinoatrial node [6]. One of the proposed mechanisms is that, during suspension laryngoscopy, the stimulus is on the laryngeal surface of the epiglottis, which differs from the vallecular stimulation by curve laryngoscopy for orotracheal intubation. Sensory innervation of the laryngeal surface of the epiglottis is mainly attributed to the internal branch of the superior laryngeal nerve, derived from the vagus nerve, and its intense stimulation may predispose asystole [7–9].

Another proposed mechanism is that cervical hyperextension may cause distension of the carotid sinus and trigger reflex in predisposed individuals, a similar event to carotid massage for the treatment of tachyarrhythmias [10].

In addition to direct activation of parasympathetic afferent fibers, the hypoxia, superficial anesthesia, or the use of vagotonic opioid drugs such as propofol and fentanyl may also contribute to bradycardia [11, 12].

**Figure 2:**
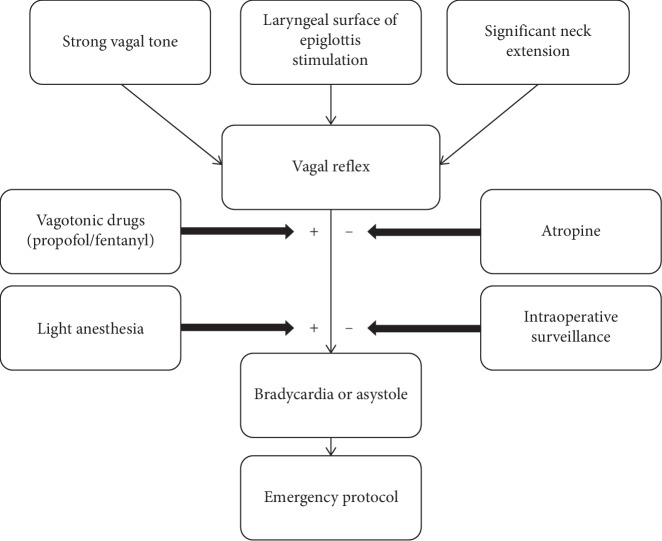
Flow chart of asystole pathophysiology during suspension laryngoscopy.

The differential diagnosis of this complication includes myocardial conduction abnormalities, pulmonary thromboembolism, stroke, or acute myocardial infarction [13]. Since the patient maintained 100% pulse oximetry throughout the procedure and all other diagnoses were excluded postoperatively, it is believed that asystole occurred due to the association of the following factors: (1) intense vagal reflex due to supraglottic manipulation in the attempt of better exposure; (2) use of vagotonic drugs; and (3) anesthesia superficialization due to the lack of stimulation for a prolonged period without use of new drugs (Figure 2).

As a prophylactic strategy, the routine use of atropine before laryngoscopy is already implemented in the pediatric population, in which intense vagal reflex is well described [14]. Adult patients submitted to suspension laryngoscopy may also benefit from the use of vagolytic drugs at the beginning of the surgical procedure, especially from high vagal tone, identified in the cardiologic evaluation [9]. As another proposal, it is suggested that deep anesthesia associated with topical lidocaine may reduce the risk of asystole, but data are still limited [15].

In addition to drug prophylaxis, constant surveillance by anesthetic and surgical staff is essential for early identification of the complication. The intense stimulation of the airway, shared by both teams, can trigger situations that require immediate solutions. Agility and good communication between surgeons, anesthesiologists, and the nursing staff are key to avoid unwanted events [16].

Laryngeal microsurgery requires the use of various equipment such as surgical microscope, recording tower, CO_2_ laser equipment, and anesthesia machine. We emphasize the importance of adopting protocols of organization of the physical space and training of the medical and paramedical teams to face emergency situations. The correct and standardized location of the equipment favors the rapid removal of the equipment and the performance of all the professionals involved in an emergency.

In addition to the good communication between the teams, the protocol of organization of the physical space standardized in our service allowed fast and safe access to the patient.

**Figure 3:**
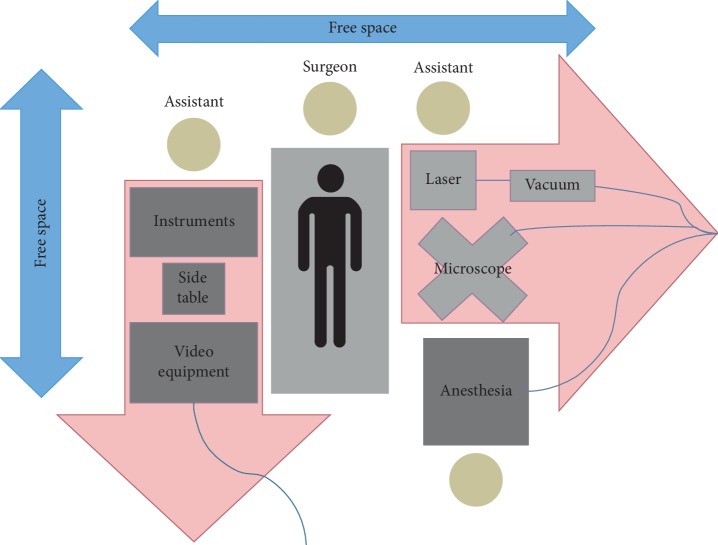
Example of the operating room map during suspension laryngoscopy.

The protocol determines the precise and constant location of all equipment and the maintenance of free spaces, with no wires or connectors around the surgical table (Figure 3). Each team member receives a specific function, which reduces the possibility of failure to assist during an emergency situation, as it ensures prompt attention to the patient.

## 4. Conclusion

Although rare, asystole secondary to direct suspension laryngoscopy is a serious complication, requiring fast and effective treatment to avoid sequelae. Drug prophylaxis is necessary, although it does not replace continuous surveillance by care teams. Systematic training based on an established protocol facilitates access and assistance to the patient in the occurrence of this complication.
